# Low-Dose Acetylsalicylic Acid in Chronic Subdural Hematomas: A Neurosurgeon's Sword of Damocles

**DOI:** 10.3389/fneur.2020.550084

**Published:** 2020-09-29

**Authors:** Lorenzo Mongardi, Flavia Dones, Giorgio Mantovani, Pasquale De Bonis, Oriela Rustemi, Luca Ricciardi, Michele Alessandro Cavallo, Alba Scerrati

**Affiliations:** ^1^Neurosurgery Department, University Hospital S. Anna, Ferrara, Italy; ^2^Department of Morphology, Surgery and Experimental Medicine, University of Ferrara, Ferrara, Italy; ^3^Neurosurgery, San Bortolo Hospital, Vicenza, Italy; ^4^Neurosurgery, Pia Fondazione di Culto e Religione Cardinal G. Panico, Tricase, Italy

**Keywords:** chronic subdural hematoma, low-dose acetylsalicylic acid, aspirin, antithrombotic drugs, cardiovascular risk, hemorrhagic risk, thromboembolic risk

## Abstract

**Background:** The possible influence of different antithrombotic drugs on outcome after neurosurgical treatment of chronic subdural hematoma (CSDH) is still unclear. Nowadays, no randomized clinical trials are available. A metanalysis including 24 studies for a total of 1,812 pooled patients concluded that antiplatelets and anticoagulations present higher risk of recurrences. On the other hand, several studies highlighted that antithrombotic suspension, timing of surgery, and resumption of these drugs are still debated, and patients taking these present higher risk of thromboembolic events with no excess risk of bleed recurrences or worse functional outcome. Our assumption is that the real hemorrhagic risk related to antithrombotic drug continuation in CSDH may be overrated and the thromboembolic risk for discontinuation underestimated, especially in patients with high cardiovascular risk.

**Methods:** A comprehensive literature review with the search terms “acetylsalicylic acid” and “chronic subdural x” was performed. Clinical status, treatment, time of drug discontinuation, complications (in particular, rebleeding or thromboembolic events), and clinical and radiological outcome at follow-up were evaluated.

**Results:** Five retrospective studies were selected for the review, three of them reporting specifically low-dose acetylsalicylic intake and two of them general antithrombotic drugs for a total of 1,226 patients. Only two papers reported the thromboembolic rate after surgery; in one paper, it is not even divided from other cardiac complications.

**Conclusion:** The literature review does not clarify the best management of low-dose acetylsalicylic in CSDH patients, in particular, concerning the balance between thromboembolic event rates and rebleeding risks. We do believe that CSDH precipitates the worsening of comorbidities with a resulting increased mortality. Further studies clearly evaluating the thromboembolic events are strongly needed to clarify this topic.

In this perspective paper, we discuss the difficult choice of low-dose acetylsalicylic acid (LDAA) management in patients suffering from chronic subdural hematoma (CSDH). The balance between hemorrhagic and thromboembolic risks often represents a sword of Damocles for neurosurgeons, especially when dealing with patients with high cardiovascular risk. No guidelines are currently available, and a survey by Kamenova et al. showed that most neurosurgeons discontinue LDAA treatment for at least 7 days in the perioperative period of surgical evacuation of CSDH, even though recent studies show that early LDAA resumption might be safe. Thrombosis prophylaxis is administered by only 60%, even though patients with CSDH are at high risk of developing thromboembolic complications. We would like to bring attention to this controversial issue.

## Introduction

The possible influence of different antithrombotic strategies on outcomes and recurrences after neurosurgical treatment of chronic subdural hematoma (CSDH) as well as resumption time of such drugs are unclear.

A new ACC/AHA guideline on the primary prevention of cardiovascular disease has recently been published (1).

While aspirin is well-established for secondary prevention of atherosclerotic cardiovascular disease (ASCVD), it should not be used in routine primary prevention due to lack of net benefit. Indeed, for decades, low-dose aspirin has been widely administered for ASCVD prevention increasing the risk of bleeding (1).

Neurosurgeons often face a difficult choice in the management of antithrombotic drugs in patients suffering from CSDHs, in terms of suspension and resumption, trying to balance the risk of hemorrhage vs. thromboembolic events (2).

Phan et al. in 2018 reported that the rate of thromboembolism was statistically lower in patients who resumed antithrombotic drugs after surgical evacuation of CSDH (2.9 vs. 6.8%, *P* < 0.001) (3). However, this paper (as reported by the authors themselves) presents several biases, including inherent patient bias in medication restrictions, selection bias in the directives from different clinicians, and attrition bias in age- and age-related comorbidities of the patients. Furthermore, other confounding factors that could affect the results are the original reason for antithrombotic treatment, the resumption time of antithrombotic agents postoperatively, and preexisting medication regimes, which were not reported in the studies included and, as such, could not be analyzed.

In this complex scenario, the individual risk of thromboembolic events and, most importantly, the kind of antithrombotic drug used needs to be seriously considered (2).

The same analysis should be performed when choosing between antiplatelet and anticoagulant drugs. As a result, an intriguing observation emerged: the disturbance in platelet function (caused almost exclusively by LDAA) correlated with improved neurological outcome at discharge. This could be influenced by the antiphlogistic properties of LDAA as well, as described by Szczygielski et al. (4), even though its effect is correlated with a higher risk to develop chronic subdural hematoma (5).

We, therefore, decided to conduct a narrative review of the literature to analyze the real influence of LDAA on outcomes after neurosurgical treatment of CSDH.

## Methods

A comprehensive review of the current literature regarding “low-dose acetylsalicylic acid” and “chronic subdural hematoma” was performed.

Using PubMed MeSH database (last search was launched in March 2020), all English papers published between the years 2000 and 2020, including the words “acetylsalicylic acid” and “chronic subdural hematoma,” were reviewed.

All papers including human participants such as randomized clinical trial, prospective or retrospective studies, as well as case reports were included, whereas articles not involving human subjects were excluded. Other reviews, editorials, and commentaries were excluded as well. Each article was scrutinized in order to select those reporting a detailed description of antiplatelet drug management, drug discontinuation, surgical vs. conservative treatment, complication rate, and a detailed clinical and radiological follow-up.

Papers reporting generic antithrombotic agents without a clear distinction between antiplatelet and anticoagulant medications were not included in the review.

## Results

**Figure 1:**
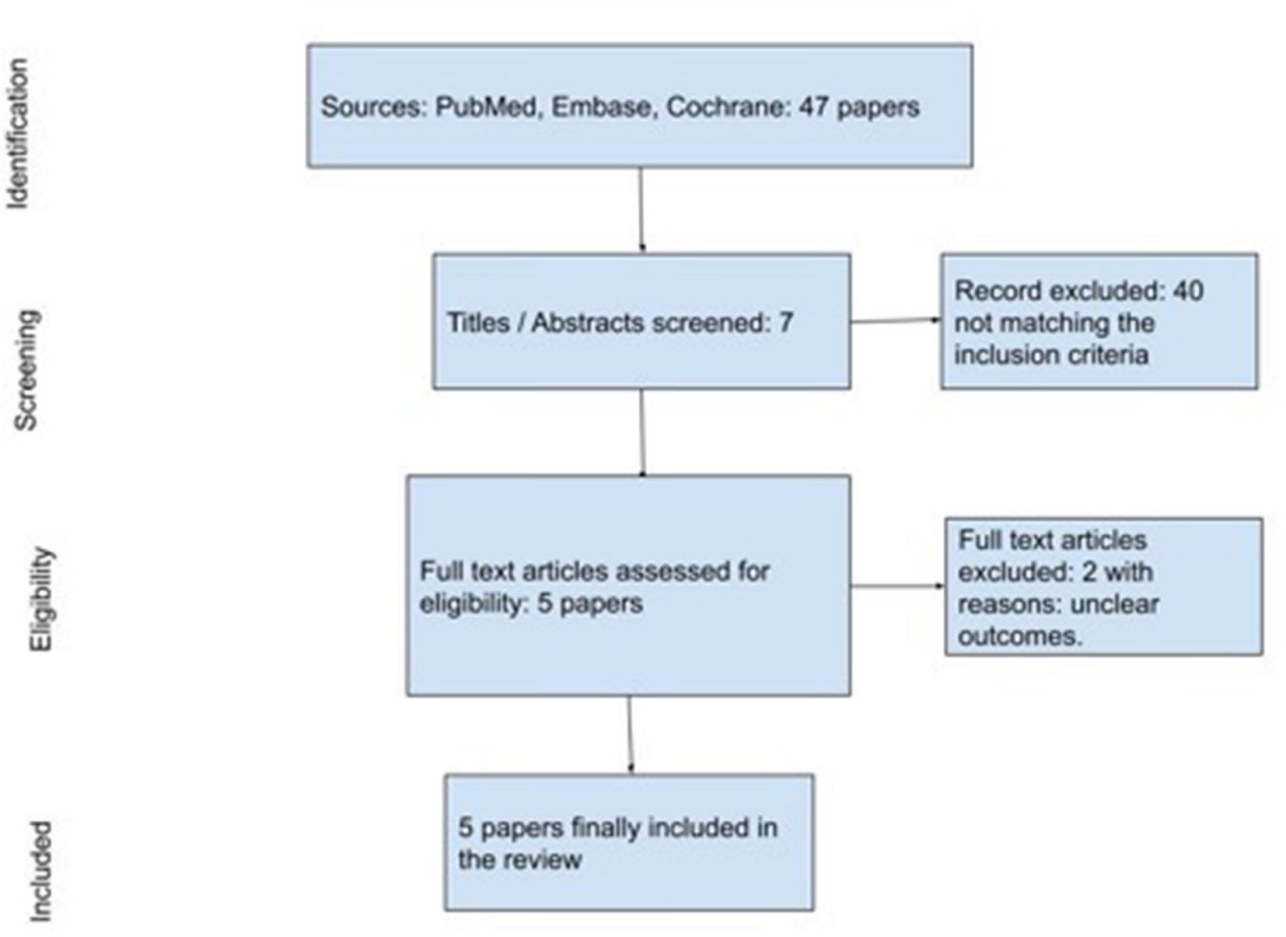
Search strategy.

From the first literature search, we retrieved 47 articles. After duplicate removal and title/abstract screening for matching inclusion/exclusion criteria, 40 papers were assessed for eligibility (Figure 1). Two papers were further excluded for the following reasons: unclear outcomes and non-elderly population.

The search strategy is reported in Figure 1.

Following the aforementioned criteria, five retrospective studies were finally selected for the review. Three studies were specifically on LDAA, while two studies reported on general antiplatelet drugs for a total amount of 1,226 patients (Table 1).

**Table 1:** Collected studies and data characteristics.

**Author/year**	**Scerrati A. 2019**	**Kamenova 2016**	**Poon 2019**	Fornebo 2017^*^	Guha 2016^*^
*N*^°^ of patients	164 patients	140 patients	135 patients	308 patients	479 patients
LDAA discontination on admission	164 patients	108 (32 patients 5 days before surgery)	135 patients	308 patients (183 were taking LDAA)	231 patients
LLDA early resumption (<48)	At 72 h (in patients at higher risk of thromboembolic complications)	At 1, 7, 14, 21, 28, 35, and 42 days after surgery, 32 patients (22.9%), the indication for LDAA treatment was not confirmed postoperatively	NN	NN	0
LDAA resumption >1 week	At least 15 days (in patients with lower cardiovascular risk)	At 1, 7, 14, 21, 28, 35, and 42 days after surgery, 32 patients (22.9%), the indication for LDAA treatment was not confirmed postoperatively	NN	100 patients (59 were taking LDAA)	120 patients
LDAA resumption >1 month	None	At 1, 7, 14, 21, 28, 35, and 42 days after surgery, 32 patients (22.9%), the indication for LDAA treatment was not confirmed postoperatively	NN	202 patients (124 started again LDAA); 6 missing patients	
Conservative treatment	None	None	None	None	
Surgical treatment	164 patients	140 patients	135 patients	308 patients	479 patients
Early surgery <5	128 patients	108 patients	NN	NN	
Late surgery >5	36 patients	32 patients	NN	NN	
CSDH early recurrence <1 week	In 20 patients, time of recurrence not specified	0 patients	10 (time of recurrence non-specified)		
CSDH late recurrence >1 week	NN	18 patients	NN	11.7% AT vs. 10.8% non-AT, 6.2% in antiaggregation group	AT restarted group, 15 patients AT non-restarted, 35 patients
Thromboembolic events	NN	Cardiovascular events: 9 (in LDAA group) and 1 (no LDAA)	4 patients	2% early group resumption 11% late group resumption (*P* < 0.01)	
Preoperative evaluation (GCS, CCI)	CCI (up to 3), 28 patients (17%) CCI (4–5), 28 patients (17%) CCI (6 or more), 108 patients (66%) The mean CCI values were 4 and 6.	NN	NN	Early group resumption/late group resumption: GCS 3–8, 6 patients (6.2)/6 patients (3.0) GCS 9–12, 9 patients (9.3)/17 patients (8.6) GCS 13–15, 82 patients (84.5)/175 patients (88.4)	
Clinical outcome	At 6 months follow-up: 51 patients (31.1%)—improvement of neurological symptoms, 80 patients (48.8%)—stable, 18 patients (11%)—worsened	NN	−38 patients—mRS 4–6 at discharge; - 54 patients—no improvement		
Mortality	15 patients	1 (LDAA group) and 4 (no LDAA)	0	4.2% AT vs. 2.4% non-AT	
Quality of the study according to NOS scale	Good	Good	Good	Good	Good

*AT, antithrombotic; LDAA, low dose acetilsalicylic acid; GCS, Glasgow coma scale; mRS, modified Rankin scale*.

* *Unspecified antiaggregation*.

° *Patients were not further classified*.

### LDAA Group

Four hundred thirty-nine patients were collected in this group. Four hundred seven (92.7%) patients discontinued LDAA on admission while 32 patients (7.3%) at least 5 days before surgery due to minor symptom presentation (6–8).

Only Kamenova et al. reported an LDAA resumption at 1, 7, 14, 21, 28, 35, and 42 days without further information. In 32 patients, the indication for LDAA treatment was not confirmed postoperatively, and the treatment was stopped (6).

All 439 patients underwent surgery: 236 patients (Scerrati et al. and Kamenova et al.) were operated before 5 days from LDAA discontinuation while 68 patients after 5 days (6, 7).

There were no cases of early recurrence (<1 week); Kamenova et al. and Poon et al. reported 28 cases of late recurrence of chronic subdural hematoma and 13 thromboembolic events after surgery. In Kamenova et al., 10 cardiovascular events comprised four myocardial infarction, one occlusion of femoral artery, three pulmonary embolisms, and two cardiac arrhythmia/decompensation, while in Poon et al., the severity of the thromboembolic events was not analyzed (6, 8).

Twenty-one patients died after surgery. Only Kamenova et al. reported the causes of death in five patients: two intraparenchymal hemorrhage after surgery, one subdural empyema, one pulmonary embolism, and one shock due to cardiopulmonary decompensation (6).

### General Antiplatelet Drug Group

General antiplatelet drugs evaluated in the abovementioned studies includes LDAA, clopidogrel, and dipyridamole (9, 10).

A total of 797 patients were included, and antiplatelet drug (AD) was discontinued on admission in 539 patients. Fornebo et al. reported that among their 308 patients, 183 were taking specifically LDAA (9).

AD therapy was resumed after >1 week in 220 patients, and after >1 month, 6 in 202 patients were lost at follow-up. Fornebo et al. reintroduced LDAA in 59 patients after 1 week and in 124 after 1 month. In 111 patients, AD therapy was not confirmed after discharge (9).

All of the 797 patients were treated surgically, but timing is not reported. No early recurrences (<1 week) were reported.

Fornebo reported late recurrences, respectively, in 36 patients (11.7%) who reintroduced AD therapy and in 33 patients (10.8%) who did not resume AD therapy. Thromboembolic events were reported on early onset in six patients (2%) and on late onset in 34 patients (11%). Mortality rate was 4.2% among patients who reintroduced the AD therapy and 2.4% in patients who discontinued it (9).

Guha reported 15 (3.1%) recurrences after >1 week in patients who reintroduced AD and 35 recurrences (7.3%) without AD therapy. No thromboembolic events and mortality are described in this series (10).

## Discussion

The real influence of different antithrombotic drugs on outcomes after neurosurgical treatment of CSDH is still unclear, especially concerning LDAA, which has widely been administered for ASCVD prevention for decades (2, 3).

In 2019, Wang et al. published a metanalysis of 24 papers with a pool of 1,812 patients on this topic, concluding that antiplatelet and anticoagulation drugs presented higher risk of recurrence in surgically treated CDSH patients (11).

On the other hand, Poon et al. and Szczygielski et al. underlined the lack of clear indication for the management of these drugs, concluding that patients on antithrombotic drugs were at higher risk of thromboembolic events with no excess risk of bleed recurrence or worse functional outcome after CSDH drainage (4, 8).

In 2013, De Bonis et al. demonstrated a significant association between antithrombotic drug intake and an increased risk of developing CSDH (12); the same group in 2018 and 2019 reported that patients on such therapy do not have an increased risk of rebleeding or worsen clinical outcome when compared with other patients (7, 13, 14).

Our literature review highlights the thromboembolic events after LDAA or antithrombotic drug suspension, and the cardiovascular risk of patients are almost always underestimated or not considered. Very few studies reported this kind of information, while most of the studies focused their attention on the rebleeding risk (6–10).

In the LDAA group, among 439 patients, LDAA was discontinued on admission in 407 patients. Only Kamenova et al. reported on LDAA resumption after surgery (6).

There were no cases of early recurrence (<1 week); Kamenova et al. and Poon et al. reported 28 cases of late recurrence and 13 thromboembolic events after surgery, without any statistically significant difference between patients who underwent discontinuation or not (6, 8).

The clinical outcome was reported in only two papers (Scerrati et al.; Poon et al.), and preoperative assessment is available only in the Scerrati et al. series, undermining any possible LDAA management/complication rate/clinical outcome comparison between the groups (7, 8).

The same limitations are present in the general antiplatelet drug group.

In this complex scenario, comorbidities, and presenting neurological conditions seem to play a significant role in the final outcome. The discontinuation of LDAA could not be absolutely necessary prior to CSDH surgery, in particular, in patients with high cardiovascular risk. Indeed, this risk is often underestimated or is not correctly stratified.

We do believe CSDH precipitates the worsening of preexisting comorbidities causing an increased mortality. In particular, in high cardiovascular risk patients, maintaining acetylsalicylic acid treatment to reduce the thromboembolic event rate could have a positive effect on the final clinical outcome.

Furthermore, the real risk of thromboembolic events after LDAA suspension for CSDH patients appears to be not well-analyzed and understood in the current literature, leaving the decision on the experience of the physician. The same issue still remains controversial in other surgery specialties, as reported by a Cochrane Systematic review (15) collecting five RCTs with 666 randomized adults and concluding that they found low-certainty evidence that either continuation or discontinuation of antiplatelet therapy before non-cardiac surgery may make little or no difference to mortality, bleeding requiring surgical intervention, or ischemic events. They also found moderate-certainty evidence that either continuation or discontinuation of antiplatelet therapy before non-cardiac surgery probably makes little or no difference in bleeding requiring transfusion.

A new approach of the clinical problem based on pre- and postsurgery clinical evaluation, with a real stratification of indications for LDAA intake and related cardiovascular risk may solve this complex dilemma.

Our perspective is to propose a clinical evaluation scale in order to stratify patient suffering from CSDH in terms of hemorrhagic and ischemic risk. This scale could be built on the basis of already validated scales (16, 17) and should comprehend different parameters. These types of scales have been widely used in general surgery (18) or cardiosurgery (19) for evaluation of the risk of postoperative thromboembolic or hemorrhagic complications (20, 21). A possible scale could be drafted as follows:

**Table:** 

**High-risk factors for thromboembolic (TE) events**	**High-risk factors for hemorrhagic events**
Previous TE event—*2 points*	Hepatic disease—*2 points*
Hypertension—*1 point*	Alcool abuse—*1 point*
Diabetes—*1 point*	Reduced platelets count or function—*2 points*
CABG grafts or previous cardiac valve surgery—*2 points*	Anemia—*1 point*
Cancer history—*1 point*	Excessive fall risk—*1 point*
Postoperative infection—*1 point*	Consistent residual subdural hematoma—*1 point*
Fibrinogen >3.5 g/L—*1 point*	Renal disease—*1 point*

According to the specific score, patients could be stratified in low, medium, or high risk for thromboembolic or hemorrhagic events, respectively, and drug management planned accordingly. In this way, there would be a numeric and objective estimation of the risks (hemorrhagic vs. thromboembolic), and the surgeon could choose “the lesser of two evils” between them.

These scores could be the first one available for neurosurgical patients, helping in the difficult decision of anticoagulant/antithrombotic management.

## Limitations

Data about the exact dosage and length of period of administration of the drugs in the collected studies are often unclear.

This is a perspective paper and not a systematic review, so we decided not to perform statistical analysis. This could constitute a bias.

One of the major limitations was the poor level of evidence of several collected studies that could represent a bias in the correct evaluation of the extracted data.

Moreover, an expected limitation of including resources with variable qualities, definitions, follow-ups, and diagnostic criteria is the inevitable heterogeneity detected in some outcomes.

## Conclusions

The literature review highlights the underestimation of the importance of thromboembolic events and cardiovascular risk of patients suffering from CSDH who are taking LDAA.

We do believe that CSDH precipitates the worsening of comorbidities causing an increased mortality. In particular, in high cardiovascular risk patients, maintaining acetylsalicylic acid treatment supposedly reduces the thromboembolic event rate and could have a positive effect on the final clinical outcome. Thus, in our opinion, the discontinuation or effect reversal for acetylsalicylic acid could not be absolutely necessary prior to CSDH surgery (particularly in patients with high cardiovascular risk).

Further studies are needed in order to clarify the role of LDAA in the management and clinical course of high cardiovascular risk patients, in particular, collecting data about thromboembolic events. An interesting perspective could be to build specific evaluation scales of risk in order to uniformly stratify this kind of patients.

## Data Availability Statement

The raw data supporting the conclusions of this article will be made available by the authors, without undue reservation.

## Ethics Statement

Ethical review and approval was not required for the study on human participants in accordance with the local legislation and institutional requirements. Written informed consent for participation was not required for this study in accordance with the national legislation and the institutional requirements.

## Author Contributions

Conception: AS and PD constructed the idea for research. Design: LM and FD designed and planned the method to achieve the results. Supervision: MC organized the execution of the study, observed the progress, and took responsibility. Data collection: OR, GM, and LR collected all the data. Analysis–interpretation: AS and PD took responsibility for the evaluation and conclusion of the findings. Literature review: OR, GM, and LR took responsibility for the literature review. Writing: LM and FD took responsibility for the writing of the entire work or its noticeable parts. Critical review: AS and MC re-evaluated the study in the scientific sense and prior to the delivery of the manuscript. All authors contributed to the article and approved the submitted version.

## Conflict of Interest

The authors declare that the research was conducted in the absence of any commercial or financial relationships that could be construed as a potential conflict of interest.

## References

[1] Arnett DK Blumenthal RS Albert MA 2019 ACC/AHA guideline on the primary prevention of cardiovascular disease: a report of the American college of cardiology/American heart association task force on clinical practice guidelines. J Am Coll Cardiol. (2019) 140:e596–646. 10.1161/CIR.0000000000000678 PMC7734661 30879355

[2] Soleman J Kamenova M Guzman R Mariani L. The management of patients with chronic subdural hematoma treated with low-dose acetylsalicylic acid: an international survey of practice. World Neurosurg. (2017) 107:778–88. 10.1016/j.wneu.2017.08.065 28838873

[3] Phan K Abi-Hanna D Kerferd J Lu VM Dmytriw AA Ho YT. Resumption of antithrombotic agents in chronic subdural hematoma: a systematic review and meta-analysis. World Neurosurg. (2018) 109:e792–9. 10.1016/j.wneu.2017.10.091 29107160

[4] Szczygielski J Utter K Oertel J. Response to poon et al. doi: 10.1089/neu.2018.6080 acetylsalicylic acid and chronic subdural hematoma: is it really a bad couple? influence of antiplatelet and anticoagulant drug use on outcomes after chronic subdural hematoma drainage. J Neurotrauma. 37:428–29. 10.1089/neu.2019.6528 31524056

[5] Connolly BJ Pearce LA Kurth T Kase CS Hart RG. Aspirin therapy and risk of subdural hematoma: meta-analysis of randomized clinical trials. J Stroke Cerebrovasc Dis. (2013) 22:444–8. 10.1016/j.jstrokecerebrovasdis.2013.01.007 23422345

[6] Kamenova M Lutz K Schaedelin S Fandino J. Does early resumption of low-dose aspirin after evacuation of chronic subdural hematoma with burr-hole drainage lead to higher recurrence rates? Neurosurgery. (2016) 79:715–21. 10.1227/NEU.0000000000001393 27538015

[7] Scerrati A Germano A Trevisi G Visani J Lofrese G. Timing of low-dose aspirin discontinuation and the influence on clinical outcome of patients undergoing surgery for chronic subdural hematoma. World Neurosurg. (2019) 129:e695–99. 10.1016/j.wneu.2019.05.252 31279757

[8] Poon MTC Rea C Kolias AG Brennan PM Influence of antiplatelet and anticoagulant drug use on outcomes following chronic subdural hematoma drainage. J Neurotrauma. (2019). 10.1089/neu.2018.6080. [Epub ahead of print]. PMC8060161 30526281

[9] Fornebo I Sjåvik K Alibeck M Kristiansson H. Role of antithrombotic therapy in the risk of hematoma recurrence and thromboembolism after chronic subdural hematoma evacuation: a population-based consecutive cohort study. Acta Neurochir. (2017) 159:2045–52. 10.1007/s00701-017-3330-x 28956170 PMC5636853

[10] Guha D Coyne S Loch MacDonald R. Timing of the resumption of antithrombotic agents following surgical evacuation of chronic subdural hematomas: a retrospective cohort study. J Neurosurg. (2015) 124:589–891. 10.1017/cjn.2015.77 26361283

[11] Wang H Zhang M Zheng H Xia X Luo K Guo F. The effects of antithrombotic drugs on the recurrence and mortality in patients with chronic subdural hematoma: A meta-analysis. Medicine. (2019) 98:e13972. 10.1097/MD.0000000000013972 30608437 PMC6344112

[12] De Bonis P Trevisi G de Waure C Sferrazza A. Antiplatelet/anticoagulant agents and chronic subdural hematoma in the elderly. PLoS ONE. (2013) 8:e68732. 10.1371/journal.pone.0068732 23874740 PMC3709887

[13] Scerrati A Mangiola A Rigoni F Olei S Santantonio M Trevisi G. Do antiplatelet and anticoagulant drugs modify outcome of patients treated for chronic subdural hematoma? Still a controversial issue. J Neurosurg Sci. (2018). 10.23736/S0390-5616.18.04311-4. [Epub ahead of print]. 29671291

[14] De Bonis P Olei S Mongardi L Cavallo MA. Chronic subdural hematoma in patients aged 80 years and older: A two-centre study. Clin Neurol Neurosurg. (2018) 170:88–92. 10.1016/j.clineuro.2018.05.002 29753169

[15] Lewis SR Pritchard MW Schofield-Robinson OJ Alderson P Smith AF. Continuation versus discontinuation of antiplatelet therapy for bleeding and ischaemic events in adults undergoing non-cardiac surgery. Cochrane Database Syst Rev. (2018) 7:CD012584. 10.1002/14651858.CD012584.pub2 30019463 PMC6513221

[16] Butchart EG Ionescu A Payne N Giddings J Grunkemeier GL Fraser AG. A new scoring system to determine thromboembolic risk after heart valve replacement. Circulation. (2003) 108(Suppl.1):II68–74. 10.1161/01.cir.0000087383.62522.1e 12970211

[17] Apostolakis S Lane DA Guo Y Buller H Lip GY. Performance of the HEMORR(2)HAGES, ATRIA, and HAS-BLED bleeding risk-prediction scores in patients with atrial fibrillation undergoing anticoagulation: the AMADEUS (evaluating the use of SR34006 compared to warfarin or acenocoumarol in patients with atrial fibrillation) study. J Am Coll Cardiol. (2012) 60:861–7. 10.1016/j.jacc.2012.06.019 22858389

[18] Fujikawa T Kawamura Y Takahashi R Naito S. Risk of postoperative thromboembolic complication after major digestive surgery in patients receiving antiplatelet therapy: lessons from more than 3,000 operations in a single tertiary referral hospital. Surgery. (2020) 167:859–67. 10.1016/j.surg.2020.01.003 32087945

[19] Reza S Pinilla N Belley-Côté EP Um KJ Sibilio S Natarajan MK. Antithrombotic management after transcatheter aortic valve replacement: a survey of Canadian physicians. Can J Cardiol. (2019) 35:1596–9. 10.1016/j.cjca.2019.08.017 31679628

[20] Park BE Bae MH Kim HJ. Perioperative outcomes of interrupted anticoagulation in patients with non-valvular atrial fibrillation undergoing non-cardiac surgery. Yeungnam Univ J Med. (2020). 10.12701/yujm.2020.00353. [Epub ahead of print]. 32668522 PMC7606955

[21] Ziviello F Pilgrim T Kroon H Ooms JF van Wiechen MP EI Azzouzi I. HAS-BLED score and actual bleeding in elderly patients undergoing transcatheter aortic valve implantation. Minerva Med. (2020) 111:203–12. 10.23736/S0026-4806.19.06154-8 32525293

