# Supplementary material for: Low-Dose Acetylsalicylic Acid in Chronic Subdural Hematomas: A Neurosurgeon's Sword of Damocles
Source: Front Neurol. 2020 Sep 29;11:550084. doi: 10.3389/fneur.2020.550084 (PMC7550681; doi:10.3389/fneur.2020.550084)
Supplement: Supplementary file 1 [file Table_1.doc]

**NEWCASTLE - OTTAWA QUALITY ASSESSMENT SCALE**

**CASE CONTROL STUDIES: FORNEBO, 2017**

Note: A study can be awarded a maximum of one star for each numbered item within the Selection and Exposure categories. A maximum of two stars can be given for Comparability.

**Selection**

1) Is the case definition adequate?

a) yes, with independent validation **🟑**

b) yes, eg record linkage or based on self reports

c) no description

2) Representativeness of the cases

a) consecutive or obviously representative series of cases **🟑**

b) potential for selection biases or not stated

3) Selection of Controls

a) community controls **🟑**

b) hospital controls

c) no description

4) Definition of Controls

a) no history of disease (endpoint) **🟑**

b) no description of source

**Comparability**

1) Comparability of cases and controls on the basis of the design or analysis

a) study controls for **recurrence of CSDH** (Select the most important factor.) **🟑**

b) study controls for any additional factor **perioperative morbidity and mortality between groups** **🟑**

**Exposure**

1) Ascertainment of exposure

a) secure record (eg surgical records) **🟑**

b) structured interview where blind to case/control status **🟑**

c) interview not blinded to case/control status

d) written self report or medical record only

e) no description

2) Same method of ascertainment for cases and controls

a) yes **🟑**

b) no

3) Non-Response rate

a) same rate for both groups **🟑**

b) non respondents described

c) rate different and no designation

**AHRQ standards: Good quality**
